# Preoperative prognostic nutritional index as an independent prognostic factor for resected ampulla of Vater cancer

**DOI:** 10.1371/journal.pone.0229597

**Published:** 2020-03-03

**Authors:** Young Jin Yoo, Chang Moo Kang, Munseok Choi, Seung Yoon Rho, Ho Kyung Hwang, Woo Jung Lee, Eun Wha Kim, Jin Ae Lee

**Affiliations:** 1 Department of Hepatobiliary and Pancreatic Surgery, Yonsei University College of Medicine, Seoul, South Korea; 2 Pancreaticobiliary Cancer Clinic, Yonsei Cancer Center, Severance Hospital, Seoul, South Korea; 3 Biostatistics Collaboration Unit, Yonsei University College of Medicine, Seoul, South Korea; Memorial University of Newfoundland, CANADA

## Abstract

**Introduction:**

Prognostic nutritional index (PNI) reflects the nutritional and immunologic status of the patients. The clinical application of PNI is already well-known in various kinds of solid tumors. However, there is no study investigating the relationship between PNI and oncological outcome of the resected ampulla of Vater (AoV) cancer.

**Materials and methods:**

From January 2005 to December 2012, the medical records of patients who underwent pancreaticoduodenectomy for pathologically confirmed AoV cancer were retrospectively reviewed. Long-term oncological outcomes were compared according to the preoperative PNI value.

**Result:**

A total of 118 patients were enrolled in this study. The preoperative PNI was 46.13±6.63, while the mean disease-free survival was 43.88 months and the mean disease-specific survival was 55.3 months. In the multivariate Cox analysis, initial CA19-9 (p = 0.0399), lymphovascular invasion (p = 0.0031), AJCC 8^th^ N-stage (p = 0.0018), and preoperative PNI (p = 0.0081) were identified as significant prognostic factors for resected AoV cancer. The disease-specific survival was better in the high preoperative PNI group (≤48.85: 40.77 months vs. >48.85: 68.05 months, p = 0.0015). A highly accurate nomogram was developed based on four clinical components to predict the 1, 3, and 5-year disease-specific survival probability (C-index 0.8169, 0.8426, and 0.8233, respectively).

**Conclusion:**

In resected AoV cancer, preoperative PNI can play a significant role as an independent prognostic factor for predicting disease-specific survival.

## Introduction

Primary ampulla of Vater (AoV) cancer only occurs in 4 to 6 cases per million population, but it is responsible for 20% of all tumor-related obstructions of the common bile duct. The incidence of this cancer has increased over the last 30 years [1, 2]. Patients undergoing pancreaticoduodenectomy (PD) for AoV cancer have a 5-year disease-free survival of approximately 65%, and the 5-year disease-specific survival varies from 33.3% to 59.9% [3–6]. These results indicate a better prognosis than that in other types of periampullary cancers. As far as recent studies are concerned, the independent factors deciding AoV cancer outcomes are AJCC T/N staging [3–6], R-status [3, 4], tumor differentiation [1, 3–5], pathological tumor size [1, 5], Different histopathologic [7], perineural invasion [8], tumor budding [9] and extranodal extension of nodal metastasis [10].

Prognostic nutritional index (PNI) is an indicator of the nutritional and immunologic status of the patients [11, 12]. Recently, multiple studies have demonstrated its correlation with postoperative complications and cancer outcomes in various kinds of solid organ cancer, such as gastric cancer [13], small cell lung cancer [14], non-small cell lung cancer [15], ovarian cancer [12], pancreatic cancer [16, 17], colorectal cancer [18], hepatocellular carcinoma [19–21], esophageal cancer [22], and renal cell carcinoma [23]. However, there is no specific study investigating the potential relationships between PNI and AoV cancer. Therefore, in this study, we investigated the potential oncological impact of preoperative PNI on resected AoV cancer.

## Materials and methods

### Patients

This was a retrospective study involving patients who underwent PD at Severance Hospital, Seoul, Korea, between January 2005 and December 2012. Only patients with pathologically confirmed AoV adenocarcinoma were enrolled in our study. The medical records and demographic characteristics of the patients were retrospectively reviewed from the electrical medical record (EMR). All data were fully anonymized before assessment and were kept on saving materials under restricted access for only authorized clinicians. The present study has waived the requirement for informed consent because of minimal risk (level I) and approved by the institutional review board (IRB) of Severance Hospital at Yonsei University College of Medicine (IRB no. 4-2019-0379).

### Preoperative and intraoperative measurements

Data on initial CA 19–9, initial total bilirubin, preoperative total bilirubin, albumin, lymphocyte count, and liver functions were collected and each PNI was calculated from the preoperative results [albumin (g/dL) × 10 + preoperative lymphocytic count × 0.005] [11]. In our study, adjusted preoperative CA 19–9 (serum CA 19–9 divided by serum total bilirubin) were applied as CA 19–9 level could be elevated from biliary obstruction, which could be helpful to reduce bias and to estimate true value of CA 19–9 [24–26].

Radiological tumor size and preoperative biliary drainage procedure were checked as each of them was known as associated with postoperative surgical outcome [27, 28]. The operation type, operation time, intraoperative estimated blood loss, and transfusion history were reviewed as covariates.

More than 90% of the patients had the operation in 6wks from the first diagnosis, and the average time from diagnosis to the operation was 18.5 days. During this period patients went further cancer evaluation and were treated for preoperative general conditions like jaundice or cholangitis.

### Pathological and postoperative outcomes

Data regarding the pathological tumor size, total number of retrieved lymph nodes, number of metastatic lymph nodes, AJCC 8^th^ TNM, perineural invasion, lymphovascular invasion, tumor differentiation, tumor gross type, R-status, and histological types were collected and examined by pathologists. The details of postoperative complications severe than Clavien-Dindo grade IIIa, postoperative pancreatic fistula (POPF) [29, 30], and adjuvant chemotherapy were collected. Adjuvant chemotherapy was selectively done under clinician’s decision in patients with advanced stages like AJCC 8^th^ T stage higher than T2, positive lymph nodes, R1 resection, or positive perineural invasion. Long-term oncological outcomes were investigated, including disease-free survival (the duration after the pancreaticoduodenectomy to the date of diagnosis of recurrent AoV cancer) and disease-specific survival (the duration from the pancreaticoduodenectomy to the time of death from AoV cancer).

### Statistical analyses

The continuous variables were expressed as the mean ± standard deviation, and the categorical variables were expressed as the frequency (%). Student’s t-test was performed with the continuous variables which were normally distributed, and Mann-Whitney U test used for the continuous variables which were not normally distributed. Chi-square test or Fisher’s extract test was used for the categorical variables.

To evaluate oncologic outcomes and survival analysis, the selection of statistically significant variables (p<0.05) was done, following univariate Cox regression test. These variables underwent multivariate Cox regression analysis to evaluate oncologic outcomes. Backward elimination used for final multivariate Cox regression results. Also Kaplan Meier survival analysis and log-rank test methods were used for survival analysis. [31–33]

For evaluating the discrimination of the predictive model, Harrell’s c-index was used for the nomogram model. The c-index and 95% confidence interval (CI) were shown after 10,000 times of bootstrap resampling. The proximity between the estimated and actual value was visually inspected with a calibration plot. The goodness of fit test was performed with GND (Greenwood-Nam-D’Agostino) test [34]. The cut-off value of PNI and CA 19–9 were calculated based on the Contal and O’quigley’s method [35–37]. SPSS Statistics version 23 was used for the analyses.

## Results

### General characteristics of the patients

A total of 118 patients were included in the study. Table 1 describes the demographic characteristics of the patients (Table 1). There were 64 males and 54 females with a mean age of 61.1 ± 10.2 years. The mean follow-up period was of 53.3 ± 34.3 months. A total of 69 patients (58.5%) survived, 49 patients (41.5%) died, and 77 patients (65.3%) received postoperative adjuvant chemotherapy.

**Table 1:** Demographic characteristics of the patients.

**Total**	N = 118 (%)
**Gender**	
Male	N = 64 (54.2)
Female	N = 54 (45.8)
**Age (Year)**	61.13 ± 10.23
**Follow-up (Month)**	53.3 ± 34.31
**Initial CA 19–9 (U/mL) (Adjusted)**	499.41 ± 2147.24
**Initial T.bil (mg/dL)**	4.45 ± 5.40
**Pre-OP T.bil (mg/dL)**	1.70 ± 1.78
**Pre-OP CEA (ng/mL)**	2.44 ± 2.36
**Pre-OP Bile drainage**	N = 70 (59.3%)
PTBD	N = 12 (17.1%),
ENBD	N = 5 (7.1%),
ERBD	N = 51 (72.9%)
Dual manner*	2 (2.9%)
**OP method**	
Open	N = 109 (92.4%)
Laparoscopic	N = 9 (7.6%)
**Subtype**	
Pancreatobiliary type	N = 63 (53.4%)
Intestinal type	N = 55 (46.6%)
**Pathological T-stage**	
Tis	N = 2 (1.7%)
1a/1b	N = 7/30 (5.9%/25.4%)
2	N = 32 (27.1%)
3a/3b	N = 19/28 (16.1%/23.7%)
**Pathological N-stage**	
N0	N = 76 (64.4%)
N1	N = 23 (19.5%)
N2	N = 19 (16.1%)
**Survival**	
Survival	N = 69 (58.5%)
Death	N = 49 (41.5%)
**Adjuvant Chemotherapy**	
Yes	N = 77 (65.3%)
No	N = 41 (34.7%)

CA 19–9, carbohydrate antigen 19–9; OP, operation; CEA, carcinoembryonic antigen; PTBD, percutaneous transhepatic biliary drainage; ENBD, endoscopic nasobiliary drainage; ERBD, endoscopic retrograde bile drainage

*PTBD followed by ERBD, ERBD followed by ENBD each.

**Fig 1:**
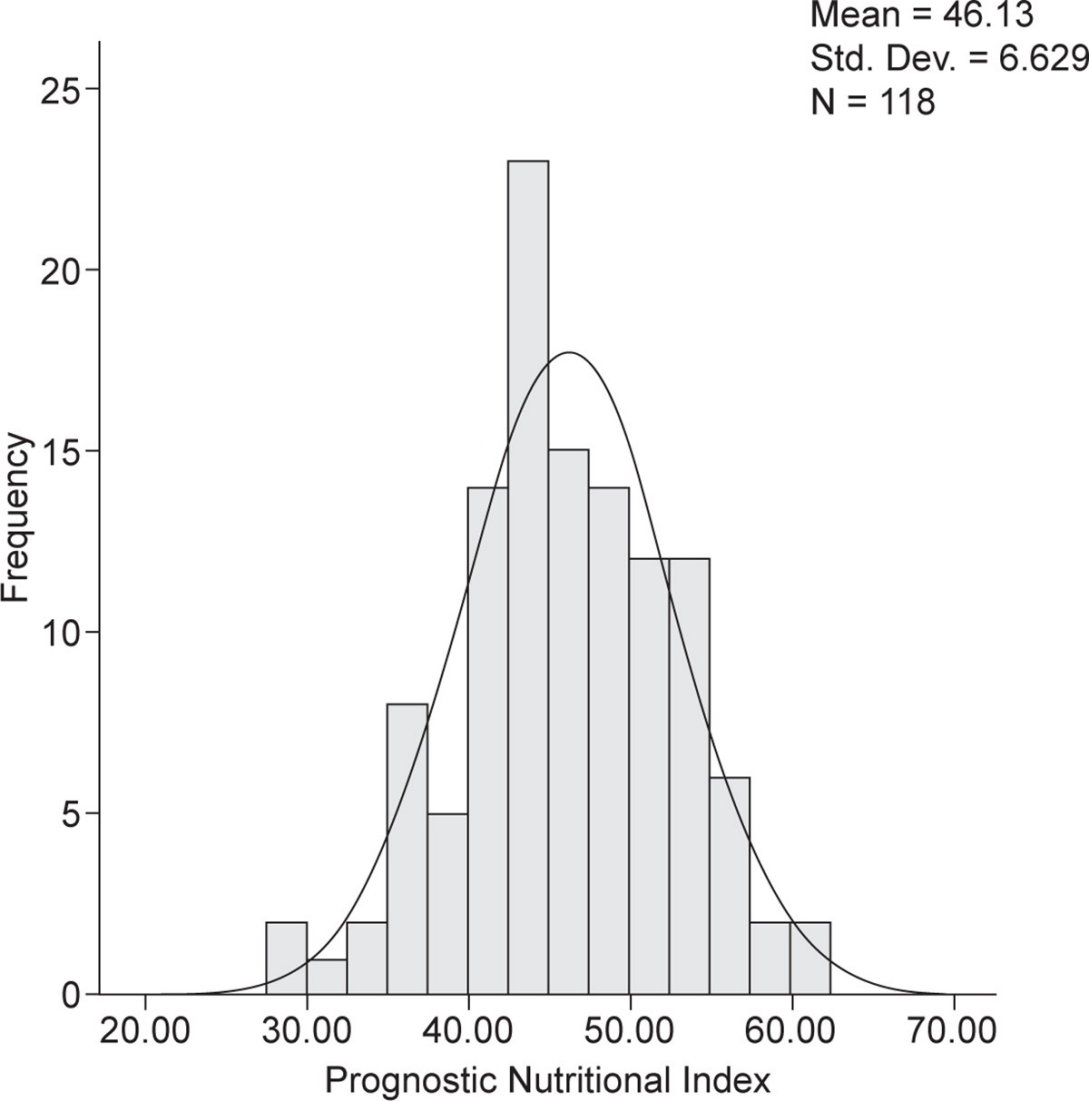
Distribution of the preoperative PNI in resected AoV cancer.

Fig 1 describes the distribution of preoperative PNI in the resected AoV cancer. The PNI was 46.13 ± 6.63 (median, 45.8) (Fig 1).

### Survival analysis in resected AoV cancer

The mean disease-free survival was found as 43.88 months, (95% CI, 38.49−49.27) and the mean disease-specific survival was 55.3 months (95% CI, 50.53−60.05). In disease-specific survival, Table 2 shows the baseline characteristics and univariable Cox regression analysis for predicting cancer-related death in the resected AoV cancer (Table 2). The left side of the table shows baseline characteristics. Student’s t-test was done in continuous variables which fit normal distribution and Mann-Whitney U test was done in continuous variables which didn’t fit normal distribution. Categorical variables were analyzed with Chi-square test or Fisher’s exact test (Table 2, Left side of the table). Univariable Cox regression analysis was done in these variables (Table 2, Right side of the table).

**Table 2:** Baseline characteristics and univariate Cox regression analysis for predicting cancer-related death in resected AoV cancer. (Left column: Baseline characteristics, Right column: univariate Cox regression analysis).

Variables				Survival N = 77	Death N = 41	p-value	HR	Lower	Upper	p-value
**Age**				62 (54 −67)	66 (57 −70)	0.0747	1.031	0.996	1.067	0.0835
**Gender**	Male			40 (51.95)	24 (58.54)	0.4940	1 (ref)			
	Female			37 (48.05)	17 (41.46)		0.745	0.400	1.386	0.3526
**BMI (kg/m**^**2**^**)**				23.44±2.91	22.95±2.5	0.3689	0.941	0.844	1.050	0.2766
Initial CA19-9 (U/mL) (cut-off)^**^ **(Adj.)** ^$ $^			<53.19	55 (71.43)	17 (42.46)	0.0015*	1 (ref)			
			≥53.19	22 (28.57)	24(58.54)		1.954	10.31	3.701	0.0399*
**Initial total bilirubin (mg/dL)**				1.50 (0.60 −4.10)	5.30 (1.60 −10.60)	0.0003*	1.079	1.041	1.118	< .0001*
**PreOP total bilirubin (mg/dL)**				0.80 (0.50 −1.80)	1.60 (0.90 −2.70)	0.0028*	1.140	1.006	1.292	0.0394*
**Radiologic tumor size (mm)**				20 (14 −25)	20 (15 −25)	0.4808	0.997	0.966	1.029	0.8511
PreOP-PNI (cut-off)^**^		PNI ≤48.85		45 (58.44)	35 (85.37)	0.0029*	1 (ref)			
		PNI >48.85		32 (41.56)	6 (14.63)		0.270	0.113	0.643	0.0031*
**PreOP-biliary drainage**		No		36 (46.75)	12 (29.27)	0.0656	1 (ref)			
		Yes		41 (53.25)	29 (70.73)		2.031	1.036	3.983	0.0392*
**Operation method**		Open		69 (89.61)	40 (97.56)	0.1595	1 (ref)			
		Lapa		8 (10.39)	1 (2.44)		0.226	0.031	1.648	0.1425
**Operation time (min)**				390 (328 −460)	409 (362 −487)	0.3268	1.002	0.999	1.004	0.2501
**Estimated blood loss (ml)**				400 (200 −700)	500 (200 −900)	0.3865	1.000	1.000	1.001	0.1483
**Transfusion**		No		63 (81.82)	32 (78.05)	0.6226	1 (ref)			
		Yes		14 (18.18)	9 (21.95)		1.034	0.493	2.167	0.9295
**Number of total retrieved LNs**				19 (11 −29)	19 (11 −28)	0.9459	1.001	0.979	1.022	0.9559
**Number of positive LNs**				0 (0 −0)	2 (0 −4)	<0.0001*	1.212	1.129	1.302	< .0001*
**Pathologic tumor size (mm)**				20 (15 −27)	20 (17 −25)	0.6931	0.995	0.966	1.024	0.7211
**Complication**		No		30 (38.96)	11 (26.83)	0.1876	1 (ref)			
		Yes		47 (61.04)	30 (73.17)		1.619	0.811	3.231	0.1719
**POPF**		No		45 (58.44)	24 (58.54)	0.0841	1 (ref)			
		Grade A		22 (28.57)	6 (14.63)		0.554	0.226	1.356	0.1958
		Grade B		10 (12.99)	10 (24.39)		1.811	0.865	3.792	0.1151
		Grade C		0 (0)	1 (2.44)		2.133	0.288	15.787	0.4583
**R-status**		R0		75 (97.4)	39 (95.12)	0.6092	1 (ref)			
		R1		2 (2.6)	2 (4.88)		2.175	0.522	9.059	0.2856
**Perineural invasion**		No		71 (92.21)	27 (65.85)	0.0003*	1 (ref)			
		Yes		6 (7.79)	14 (34.15)		3.141	1.640	6.017	0.0006*
**Lymphovascular invasion**		No		66 (85.71)	25 (60.98)	0.0023*	1 (ref)			
		Yes		11 (14.29)	16 (39.02)		3.758	1.993	7.088	< .0001*
**Subtype**		PB		34 (44.16)	29 (70.73)	0.0059*	1 (ref)			
		Int.		43 (55.84)	12 (29.27)		0.341	0.173	0.671	0.0018*
**Gross type**		Polypoid		51 (66.23)	27 (65.85)	0.8411	1 (ref)			
		Ulcerative		14 (18.18)	7 (17.07)		0.922	0.403	2.110	0.8481^$^
		Mixed		2 (2.6)	0 (0)		0.918	0.052	16.154	0.9531^$^
		Unknown		10 (12.99)	7 (17.07)		1.369	0.599	3.132	0.4565^$^
**Tumor grade**		Well		34 (44.16)	8 (19.51)	0.0176*	1 (ref)			
		Moderate		40 (51.95)	30 (73.17)		2.733	1.249	5.978	0.0118*
		Poor		3 (3.9)	3 (7.32)		7.286	1.900	27.942	0.0038*
**T-stage (AJCC 8**^**th**^**)**		IA		8 (10.39)	1 (2.44)	0.0217*	1 (ref)			
		IB		25 (32.47)	5 (12.2)		1.743	0.204	14.920	0.6121
		II		20 (25.97)	12 (29.27)		4.469	0.581	34.382	0.1504
		IIIA		11 (14.29)	8 (19.51)		5.288	0.661	42.321	0.1166
		IIIB		13 (16.88)	15 (36.59)		8.274	1.090	62.793	0.0410*
**N-stage (AJCC 8**^**th**^**)**		N0		60 (77.92)	16 (39.02)	0.0001*	1 (ref)			
		N1		9 (11.69)	14 (34.15)		4.335	2.099	8.954	< .0001*
		N2		8 (10.39)	11 (26.83)		7.770	3.518	17.159	< .0001*
**Postop-adjuvant chemotherapy**		No		52 (67.53)	16 (39.02)	0.0028*	1 (ref)			
		Yes		25 (32.47)	25 (60.98)		2.303	1.227	4.324	0.0094*
**Recurrence**		No		62 (80.52)	7 (17.07)	< .0001*	1 (ref)			
		Yes		15 (19.48)	34 (82.93)		14.558	6.244	33.943	< .0001*

HR, hazard ratio; BMI, body mass index; PreOP, preoperative; LN, lymph node; PNI, prognostic nutritional index; POPF, postoperative pancreatic fistula; PB, pancreatobiliary type; Int., intestinal type.

*p-value <0.05

**Cut off value deducted from the Contal and O’quigley’s method [35–37].

^$^Using firth bias correction for the estimation of 95% CI [38].

^$ $^Adj. = Adjusted

Among the preoperative factors, adjusted initial CA19-9 of ≥53.19 (p = 0.0015), initial/preoperative total bilirubin (p = 0.0003, p = 0.0028 respectively), and preoperative PNI of ≤48.85 (p = 0.0029) were noted as significant variables. The number of positive metastatic lymph node was found as a significant variable among the intraoperative factor in predicting the survival (p<0.0001). Among the postoperative factors, it was found that perineural invasion (p = 0.0003), lymphovascular invasion (p = 0.0023), subtype of tumor (p = 0.0059), tumor grade (p = 0.0176), AJCC 8^th^ T/N-stage (p = 0.0217, p = 0.0001 each), postoperative adjuvant chemotherapy (p = 0.0028), and recurrence (p = <0.0001) were significant [1, 3–6].

### Preoperative PNI as an independent prognostic factor

Multivariate Cox analysis was used to predict the significant prognostic factors in resected AoV cancer (Table 3). Adjusted initial CA 19–9 [HR = 1.954 (95% CI, 1.031−3.701), p = 0.0399], lymphovascular invasion [HR = 2.775 (95% CI, 1.412−5.452), p = 0.0031], AJCC 8^th^ N stage [N-Stage 1: HR = 3.282 (95% CI, 1.553−6.932), p = 0.0018; N-Stage 2: HR = 4.978 (95% CI, 2.122−11.676), p = 0.0002, respectively], and PreOP-PNI [HR = 0.300 (95% CI, 0.123−0.732), p = 0.0081] were identified as important factors for disease-specific survival in resected AoV cancer.

**Table 3:** Multivariate Cox analysis for predicting the disease-specific survival in resected AoV cancer.

Variables		Death (0: survival, 1: death)			
		HR	Lower	Upper	p-value
**Initial CA19 (U/mL) (Adjusted)**	0: CA19 <53.19	1 (ref)			
	1: CA19 ≥53.19	1.954	1.031	3.701	**0.0399**
**Lymphovascular invasion**	0: No	1 (ref)			
	1: Yes	2.775	1.412	5.452	**0.0031**
**AJCC8_Nstage**	0: No	1 (ref)			
	1: N1	3.282	1.553	6.932	**0.0018**
	2: N2	4.978	2.122	11.676	**0.0002**
**PreOP-PNI**	0: PNI ≤48.85	1 (ref)			
	1: PNI >48.85	0.300	0.123	0.732	**0.0081**

**Fig 2:**
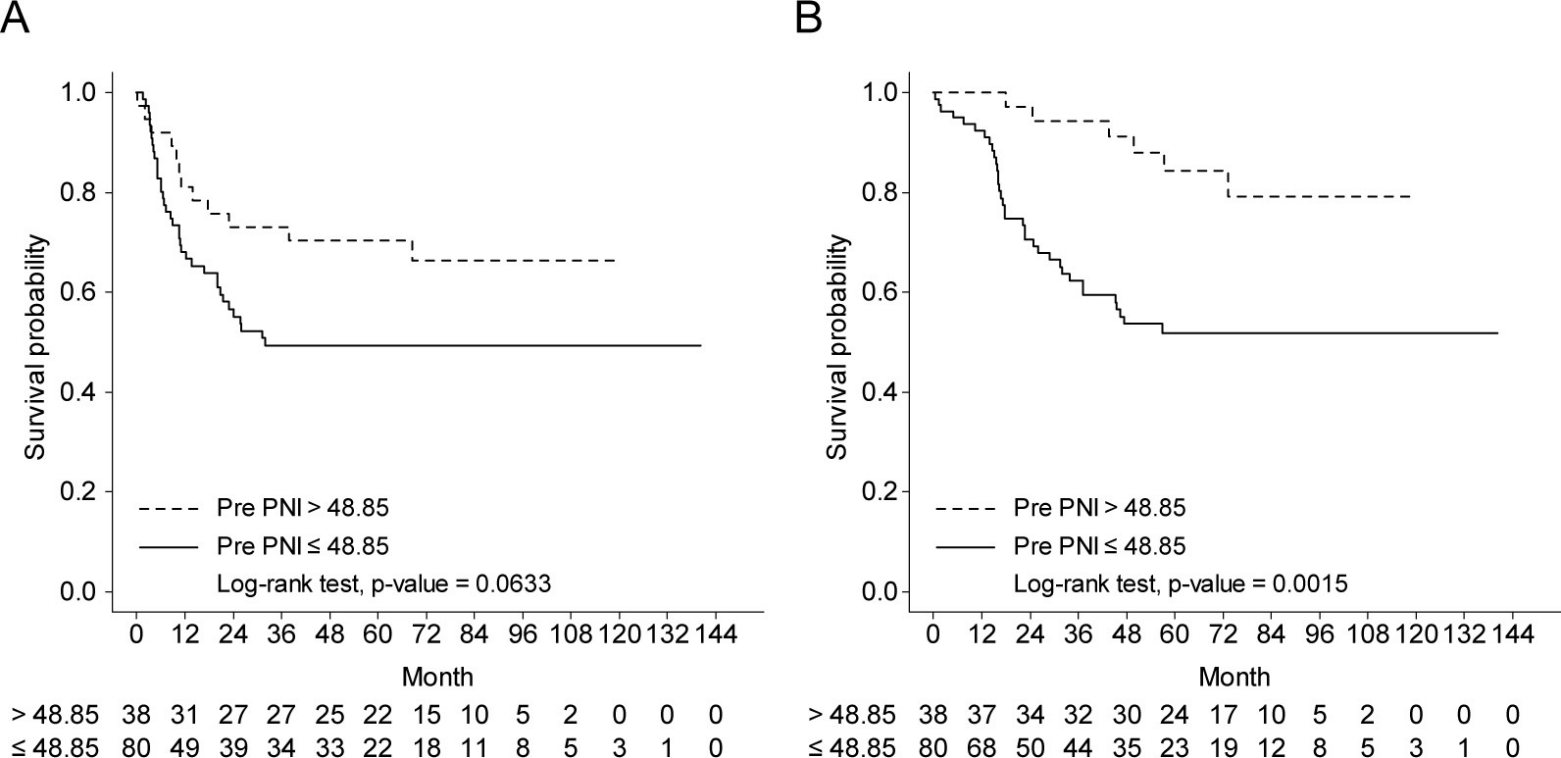
Long term oncological outcomes according to the preoperative PNI in resected AoV cancer. (A) Disease-free survival. (B) Disease-specific survival.

It was estimated that the disease-free survival was different according to the preoperative PNI with a marginal significance [≤48.85: 21.75 months (95% CI, 19.03−24.4) vs. >48.85: 51.88 months (95% CI, 43.10−60.66), p = 0.0633, Fig 2A]. However, significant difference showed in disease-specific survival according to preoperative PNI [≤48.85: 40.77 months (95% CI, 36.28−45.26), vs. <48.85: 68.05 months (95% CI, 63.02−73.06) p = 0.0015, Fig 2B].

Survival analyses at PNI high/low group under stratification was done to evaluate if PNI correlates with disease status and to exclude bias from our study (Table 4.). However it showed no difference of important factors for disease-specific survival in resected AoV cancer even after stratify with high/low PNI group (Initial CA19-9, Lymphovascular invasion, AJCC 8^th^ N stage at common) (Table 5.).

**Table 4:** Univariate Cox analysis for predicting the disease-specific survival in resected AoV cancer. (PNI group stratification).

Variables		Low group N = 80	High group N = 38	p-value	HR	Lower	Upper	p-value
**Age**		62 (56–69)	59 (51–66)	0.034*	1.019	0.985	1.055	0.279
**Gender**	Male	47 (58.8)	17 (44.7)	0.219	1 (ref)			
	Female	33 (41.3)	21 (55.3)		0.883	0.504	1.548	0.664
**BMI (kg/m**^**2**^**)**		22.86±2.65	24.14±2.85	0.018*	0.973	0.870	1.089	0.639
Initial CA19-9 (U/mL) (cut-off)^**^ **(Adj.)** ^$ $^	<53.19			42(52.5)	30(78.9)	0.011*	1 (ref)			
	≥53.19	38(47.5)	8(21.1)		2.643	1.503	4.649	0.001*
**Initial total bilirubin (mg/dL)**		5.52 (1.67–9.37)	2.21 (1.31–3.11)	<0.001*	1.062	1.023	1.102	0.002*
**PreOP total bilirubin (mg/dL)**		2.05 (1.00–3.10)	0.97 (0.67–1.27)	0.001*	1.035	0.938	1.227	0.304
**Radiologic tumor size (mm)**		21 (16–26)	20 (15–26)	0.568	0.995	0.963	1.028	0.761
**PreOP-biliary drainage**	No	24(30.0)	24(63.2)	0.001*	1 (ref)			
	Yes	56(70.0)	14(36.8)		1.453	0.812	2.598	0.208
**Operation method**	Open	77(96.3)	32(84.2)	0.030*	1 (ref)			
	Lapa	3(3.8)	6(15.8)		0.183	0.025	1.323	0.092
**Operation time (min)**		406 (356–457)	423 (347–500)	0.584	1.002	0.999	1.004	0.183
**Estimated blood loss (ml)**		550 (256–844)	538 (263–813)	0.906	1.000	1.000	1.001	0.137
**Transfusion**	No	62(77.5)	33(86.8)	0.343	1 (ref)			
	Yes	18(22.5)	5(13.2)		1.017	0.520	1.991	0.960
**Number of total retrieved LNs**		23 (15–31)	20 (9–31)	0.235	1.000	0.978	1.023	0.971
**Number of positive LNs**		2 (1–4)	0 (0–0)	0.132	1.183	1.101	1.271	<0.001*
**Pathologic tumor size (mm)**		23 (18–28)	22 (15–29)	0.768	0.992	0.963	1.021	0.584
**Complication**	No	32(40.0)	9(23.7)	0.125	1 (ref)			
	Yes	48(60.0)	29(76.3)		1.128	0.626	2.032	0.688
**POPF**	No	53(66.3)	16(42.1)	<0.001*	1 (ref)			
	Grade A	9(11.3)	19(50.0)		0.817	0.398	1.677	0.582
	Grade B	18(22.5)	2(5.3)		1.379	0.652	2.916	0.400
	Grade C	0(0)	1(2.6)		3.635	0.487	27.127	0.208
**R-status**	R0	76(95.0)	38(100.0)	0.304	1 (ref)			
	R1	4(5.0)	0(0)		2.727	0.842	8.834	0.094
**Perineural invasion**	No	64(80.0)	34(89.5)	0.308	1 (ref)			
	Yes	16(20.0)	4(10.5)		2.584	1.403	4.762	0.002*
**Lymphovascular invasion**	No	59(73.8)	32(84.2)	0.303	1 (ref)			
	Yes	21(26.2)	6(15.8)		2.531	1.386	4.621	0.003*
**Subtype**	PB	49(61.2)	14(36.8)	0.022*	1 (ref)			
	Int.	31(38.8)	24(63.2)		0.226	0.115	0.444	<0.001*
**Gross type**	Polypoid	48(60.0)	30(79.0)	0.255	1 (ref)			
	Ulcerative	17(21.3)	4(10.5)		2.200	0.828	5.846	0.114 $
	Mixed	2(2.5)	0(0)		2.000	0.120	33.270	0.629 $
	Unknown	13(16.2)	4(10.5)		3.667	1.220	11.021	0.021 $
**Tumor grade**	Well	26(32.5)	16(42.1)	0.535	1 (ref)			
	Moderate	50(62.5)	20(52.6)		3.333	1.547	7.181	0.002*
	Poor	4(5.0)	2(5.3)		9.910	32.05	30.639	<0.001*
**T-stage (AJCC 8**^**th**^**)**	IA	4(5.0)	5(13.2)	0.120	1 (ref)			
	IB	18(22.5)	12(31.6)		15786.3	0.000	1.246 E+75	0.908
	II	20(25.0)	12(31.6)		27052.2	0.000	2.134 E+75	0.902
	IIIA	16(20.0)	3(7.8)		48475.2	0.000	3.825 E+75	0.897
	IIIB	22(27.5)	6(15.8)		89926.6	0.000	7.092 E+75	0.891
**N-stage (AJCC 8**^**th**^**)**	N0	49(61.2)	27(71.1)	0.247	1 (ref)			
	N1	15(18.8)	8(21.1)		4.281	2.141	8.563	<0.001*
	N2	16(20.0)	3(7.8)		9.764	4.879	19.540	<0.001*
**Postop-adjuvant chemotherapy**	No	42(52.5)	27(71.1)	0.087	1 (ref)			
	Yes	38(47.5)	11(28.9)		2.502	1.406	4.453	0.002*

HR, hazard ratio; BMI, body mass index; PreOP, preoperative; LN, lymph node; PNI, prognostic nutritional index; POPF, postoperative pancreatic fistula; PB, pancreatobiliary type; Int., intestinal type.; E+, exponential

*p-value <0.05

**Cut off value deducted from the Contal and O’quigley’s method [35–37].

^$^Using firth bias correction for the estimation of 95% CI [38].

^$ $^Adj. = Adjusted

**Table 5:** Multivariate Cox analysis for predicting the disease-specific survival in resected AoV cancer (PNI group stratification).

Variables		Death (0: survival, 1: death)			
		HR	Lower	Upper	p-value
**Initial CA19 (Adjusted) (U/mL)**	0: CA19 <53.19	1 (ref)			
	1: CA19 ≥53.19	2.058	1.073	3.937	**0.030**
**Lymphovascular invasion**	0: No	1 (ref)			
	1: Yes	2.703	1.372	5.319	**0.004**
**AJCC8_Nstage**	0: No	1 (ref)			
	1: N1	3.341	1.584	7.047	**0.002**
	2: N2	4.828	2.062	11.308	**<0.001**

Statistical analysis of disease free-survival is noted on S1 and S2 File. S2 File is the analysis of disease free survival after PNI stratification. S1 File showed that adjusted CA 19–9, preoperative T.bilirubin positive lymph node number and subtype of adenocarcinoma were relevant with oncologic survival. S2 File showed adjusted initial CA 19–9, the subtype of adenocarcinoma, and AJCC 8^th^ N stage had statistical significance to disease free survival after PNI stratification.

### Developing nomogram to predict cancer-specific survival

**Fig 3:**
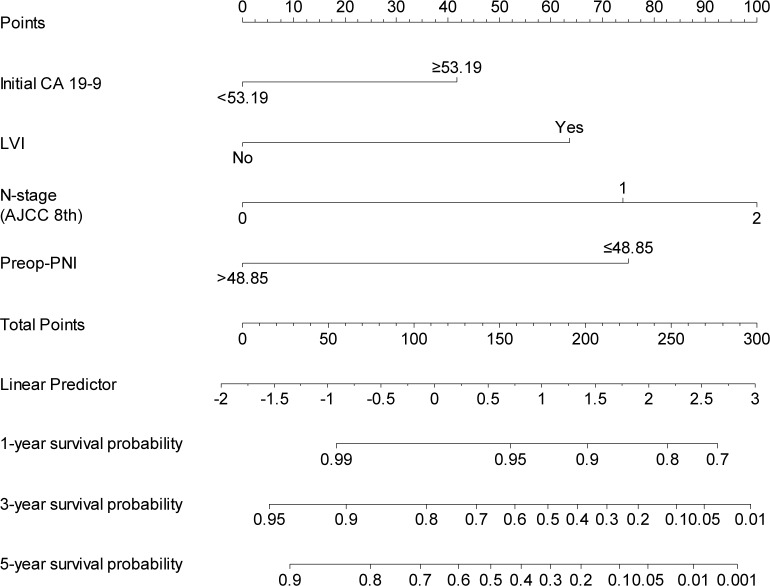
Nomogram to predict the disease-specific survival in resected AoV cancer.

Based on the significant independent variables, such as adjusted initial CA 19–9, lymphovascular invasion, AJCC N-stage, and preoperative PNI, a nomogram for predicting the 1, 3, and 5-year survival probability in resected AoV cancer was developed (Fig 3).

### Model performance and calibration

The performance of the nomogram was assessed with Harrell’s C-index (Table 6). The c-index and 95% CI were shown after 10,000 times of bootstrap resampling. Every single average C-index was noted to be >0.80 with a narrow confidence interval suggesting that the currently developed nomogram model was highly predictive.

**Table 6:** Model performance.

Overall		1-year		3-year		5-year	
C-index	95% CI	C-index	95% CI	C-index	95% CI	C-index	95% CI
0.8171	0.7558−0.8737	0.8169	0.6531−0.9643	0.8426	0.7773−0.9024	0.8233	0.7622−0.8812

**C-index:** <0.5 (very poor model), 0.5 (no better than random change), 0.7−0.8 (good model), >0.8 (strong model), 1 (perfectly predicts a certain outcome).

**Fig 4:**
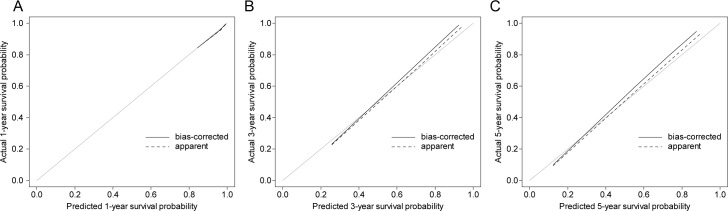
Calibration plot (A) predicted the 1-year survival probability, (B) predicted the 3-year survival probability, and (C) predicted the 5-year survival probability.

The calibration plot was made by comparing the predictive value with the real value. Considering that approximation with the 45-degree oblique dotted line estimates better results, the present calibration plot suggests that our nomogram has an acceptable accuracy in predicting the survival in resected AoV cancer (Fig 4).

## Discussion

In cancer patients, it is well known that the nutritional status is a conclusive independent factor for the postoperative outcomes [12]. In addition, nutrition correlates with general immunological functions and internal metabolisms. One commonly used indicator for nutrition is the PNI, which is calculated by using two clinical variables: preoperative albumin and lymphocytic count in the blood [11]. Recently, multiple studies have shown that preoperative PNI is a good predictive factor for estimating cancer outcome after cancer surgery [11], such as gastric cancer [13], esophageal cancer [22], hepatocellular cancer [19–21], pancreatic cancer [16, 17], colorectal cancer [18], renal cell carcinoma [23], non-small cell lung cancer [15], and small cell lung cancer [14].

TNM staging, recurrence, pathological tumor size, and tumor differentiation are the factors for predicting the postoperative oncological outcome of resected AoV cancer [1, 3–6]. Till now, no study has reported the potential oncological impact of preoperative PNI in resected AoV cancer. In this study, it has been successfully demonstrated that there is a potential association between the preoperative PNI and the long-term oncological outcome in resected AoV cancer. In this study, in the univariate analysis, the adjusted initial CA19-9 of ≥53.19, initial/preoperative total bilirubin, preoperative PNI of ≤48.85, number of positive metastatic lymph nodes, perineural invasion, lymphovascular invasion, subtype of the tumor, tumor grade, AJCC 8^th^ T/N-stage, postoperative adjuvant chemotherapy, and recurrence were identified as significant variables to predict cancer-related survival, concurrent to previous studies [3–6]. The subsequent multivariate Cox analysis found that preoperative PNI can predict the long-term survival [HR = 0.300 (95% CI, 0.123−0.732), p = 0.0081] along with other well-known clinical parameters, such as adjusted initial CA 19–9 (p = 0.0399), lymphovascular invasion (p = 0.0031), and AJCC 8^th^ N staging (p<0.05). Although there were no significant differences in the disease-free survival [Preop-PNI of ≤48.85: 21.75 months (95% CI, 19.03−24.4) vs. preop-PNI of >48.85: 51.88 months (95% CI, 43.10−60.66), p = 0.0633], it was found that the higher Preop-PNI group showed a significant positive oncological impact on the disease-specific survival in resected AoV cancer [40.77 months (95% CI, 36.28−45.26] vs. 68.04 months (95% CI, 63.02−73.06), p = 0.0015].

These results display that the potentiality of PNI is not inferior to that of the well-known predictive factors. Further, PNI could play a role as an independent factor influencing the overall survival. Without pathological confirmation, we can simply calculate PNI from the basic laboratory results and can predict only with the imaging study. This can be helpful for preoperative risk assessment and diagnosis of the hazardous group. Though it had no effect on the disease-free survival, patients who had a higher PNI of 48.85 had a significant benefit on the disease-specific survival in resected AoV cancer with an HR of 0.300 (p = 0.0081). In addition, we’ve done stratified survival analyses to evaluate if preOP PNI indicates the advanced disease stage. In multivariate Cox analysis, there was no difference of statistically significant variables in disease-specific survival in resected AoV cancer (Adjusted initial CA19-9 HR 0.486, p = 0.030; Lymphovascular invasion HR 2.703, p = 0.004; AJCC 8^th^ N stage N1-HR 3.341, p = 0.002 / N2-HR 4.828, p<0.001).

In addition, preoperative a PNI-based nomogram was developed to calculate the postoperative long-term oncological outcomes in resected AoV cancer. Predicting the power assessed with Harrell’s C-index showed PNI-based nomogram works well, and the survival probability at 1-year, 3-year, and 5-year showed the C-index of >0.80 with a short 95% CI range.

As mentioned before, the most well-known prognostic factors for resected AoV cancer are mostly determined based on pathological examination after surgical excision. However, surgeons or clinicians can easily calculate the preoperative PNI from routine blood laboratory tests. Therefore, it is anticipated that the present study can be helpful in predicting the postoperative long-term oncological outcomes clinically prior to the surgical approach. It suggests that the oncological outcomes can be modulated by surgeons or clinicians before surgery. Unlike other prognostic factors, such as adjusted initial CA 19–9, lymphovascular invasion, and N-stage, preoperative PNI is thought to be affected by patient’s general condition and nutritional status, which can be improved by appropriate preoperative management, such as nutritional support or conservative management for improving the general condition.

Although patients with AoV cancers have a typical characteristic that presents a history of obstructive jaundice, our results showed the growing type of tumor did not have much effect on preoperative PNI by chronic loss of appetite with slow-growing. (fast-growing pancreatobiliary type PNI-median 44.9, slow-growing intestinal type PNI-median 47.5, p = 0.032)

Albumin, prealbumin, and transferrin are well known prognostic factors in various kinds of solid cancer [39–46]. Albumin can be routinely checked and the impact of albumin is thought to be incorporated in the concept of PNI (albumin (g/dL)×10 + preoperative lymphocytic count×0.005). In this study, PNI is currently known as a better factor in reflecting prognosis than albumin. However, our center does not routinely check prealbumin and transferrin. Therefore, the potential oncologic impact of these factors in managing AoV cancer needs to be further investigated in near future.

Even though the exact mechanism how PNI affects cancer outcomes is not understood yet, nowadays researchers are focusing on immunity and nutritional factor [47–50]. It is hypothesized that patients with high PNI may have the appropriate general conditions, as result, they can be easily presumed to have better compliance at adjuvant treatment, which could make difference in long term oncologic outcomes. The mechanism of PNI and the way to improve preoperative PNI are potential topics to be investigated in the near future by our further studies.

Our study has several limitations. It had a retrospective study design and a limited number of patients were included. The nomogram developed also needs external validation. Further study is necessary to reconfirm the potential association between preoperative PNI and long-term oncological outcomes based on a large study population. In summary, the present study showed that preoperative PNI was an independent prognostic factor for predicting the long-term oncological outcomes in resected AoV cancer. This is the first study to show the potential oncological impact of preoperative PNI in resected AoV cancer, suggesting that improving the preoperative PNI can result in a positive oncological impact in resected AoV cancer.

## Supporting information

S1 File Statistical analysis of disease-free survival. (DOCX)

S2 File Statistical analysis of disease-free survival under PNI stratification. (DOCX)
