# Supplementary material for: Preoperative prognostic nutritional index as an independent prognostic factor for resected ampulla of Vater cancer
Source: PLoS One. 2020 Mar 3;15(3):e0229597. doi: 10.1371/journal.pone.0229597 (PMC7053754; doi:10.1371/journal.pone.0229597)
Supplement: S1 File — (DOCX) [file pone.0229597.s001.docx]

**Supplement 1. Statistical analysis of disease free survival (DFS)**

**Table 1. Baseline characteristics**

- **Cut off value deducted from the Contal and O’quigley’s method

| **Variables** | **Index level** | **0 (survival)**  **(N=69)** | **1 (recur)**  **(N=49)** | **p-value** |
| --- | --- | --- | --- | --- |
| Age (Year) |  | 60 (53 – 77) | 62 (57 – 68) | 0.229 |
| Initial CA19-9 (U/mL) |  | 55.2 (36.8 – 73.6) | 97.6 (69.6 – 125.6) | 0.065 |
| Initial T.Bilirubin (mg/dL) |  | 3.27 (1.32 – 5.22) | 6.10 (1.85 – 10.35) | 0.001 |
| Pre-OP T. bilirubin (mg/dL) |  | 1.49 (0.74 - 2.24) | 2.01 (0.96 – 3.06) | 0.016 |
| Pre-OP PNI |  | 46.91±7.05 | 45.04±5.89 | 0.120 |
| BMI |  | 23.31±2.84 | 23.22±2.70 | 0.858 |
| OP time (min) |  | 400 (330 - 470) | 428 (358 - 498) | 0.372 |
| Estimated Blood Loss (ml) |  | 508 (248 - 762) | 600 (263 - 937) | 0.226 |
| Total LN number |  | 21 (12 - 30) | 22 (11 - 33) | 0.469 |
| Positive LN number |  | 0 (0 - 0) | 3 (0 - 5) | <0.001 |
| Radiologic tumor size (mm) |  | 20 (14 - 26) | 22 (17 - 26) | 0.011 |
| Pathologic tumor size (mm) |  | 20 (14 - 26) | 24 (19 - 30) | 0.018 |
| Gender | 1: Male | 38(55.1%) | 26(53.1%) | 0.853 |
|  | 2: Female | 31(44.9%) | 23(46.9%) |  |
| Pre-OP Bile drainage | 0: No | 30(43.5%) | 18(36.7%) | 0.569 |
|  | 1: Yes | 39(56.5%) | 31(63.3%) |  |
| OP Method | 1: open | 61(88.4%) | 48(98.0%) | 0.078 |
|  | 2: lapa | 8(11.6%) | 1(2.0%) |  |
| Transfusion | 0: No | 57(82.6%) | 38(77.6%) | 0.638 |
|  | 1: Yes | 12(17.4%) | 9(22.4%) |  |
| Complication | 0: No | 24(34.8%) | 17(34.7%) | 1.000 |
|  | 1: Yes | 45(65.2%) | 32(65.3%) |  |
| POPF | 0: No | 40(58.0%) | 29(59.2%) | 0.593 |
|  | 1: Grade A | 18(26.1%) | 10(20.4%) |  |
|  | 2: Grade B | 11(15.9%) | 9(18.4%) |  |
|  | 3: Grade C | 0(0%) | 1(2.0%) |  |
| Residual Cancer Criteria | 0: R0 | 68(98.6%) | 46(93.9%) | 0.306 |
| (R-status) | 1: R1 | 1(1.4%) | 3(6.1%) |  |
| Perineural invasion | 0: No | 64(92.8%) | 34(69.4%) | 0.001 |
|  | 1: Yes | 5(7.2%) | 15(30.6%) |  |
| Lymphovascular invasion | 0: No | 58(84.1%) | 33(67.3%) | 0.045 |
|  | 1: Yes | 11(15.8%) | 16(32.7%) |  |
| Subtype of adenocarinoma | 1: pancreatobiliary | 25(36.2%) | 38(77.6%) | <0.001 |
|  | 2: intestinal | 44(63.8%) | 11(22.4%) |  |
| Tumor gross type | 1: polypoid | 52(75.4%) | 26(53.1%) | 0.048 |
|  | 2: ulceration | 10(14.5%) | 11(22.4%) |  |
|  | 3: mixed | 1(1.4%) | 1(2.0%) |  |
|  | 4: unknown | 6(8.7%) | 11(22.4%) |  |
| Tumor differentiation grade | 1: well | 34(49.3%) | 8(16.3%) | <0.001 |
|  | 2: moderate | 34(49.3%) | 36(73.5%) |  |
|  | 3: poor | 1(1.4%) | 5(10.2%) |  |
| AJCC8th Tstage | 1: Tis+IA | 9(13.0%) | 0(0%) | <0.001 |
|  | 2: IB | 23(33.3%) | 7(14.3%) |  |
|  | 3: II | 21(30.4%) | 11(22.4%) |  |
|  | 4: IIIA | 9(13.0%) | 10(20.4%) |  |
|  | 5: IIIB | 7(10.1%) | 21(42.9%) |  |
| AJCC8th Nstage | 0: No | 58(84.1%) | 18(36.7%) | <0.001 |
|  | 1: N1 | 8(11.6%) | 15(30.6%) |  |
|  | 2: N2 | 3(4.3%) | 16(32.7%) |  |
| Adjuvant CTx | 0: No | 50(72.5%) | 19(38.8%) | <0.001 |
|  | 1: Yes | 19(27.5%) | 30(61.2%) |  |
| PNI_cut off** | 0: PNI≤48.85 | 43(62.3%) | 37(75.5%) | 0.163 |
|  | 1: PNI>48.85 | 26(37.7%) | 12(24.5%) |  |
| Initial_CA19_cut off** | 0: initial_CA19-9<53.19 | 49(71.0%) | 23(46.9%) | 0.012 |
|  | 1: initial_CA19-9≥53.19 | 20(29.0%) | 26(53.1%) |  |

**Table 2. Univariable Cox regression result**

- **Cut off value deducted from the Contal and O’quigley’s method
- $: firth bias correction to regress 95% CI of HR.

| **Variables** | **Index level** | **Death (0: survival 1: recur)** | | | |
| --- | --- | --- | --- | --- | --- |
|  |  | **HR** | **Lower** | **Upper** | **p-value** |
| Age (Year) |  | 1.018 | 0.989 | 1.049 | 0.229 |
| Initial CA19-9 (U/mL) |  | 1.000 | 1.000 | 1.000 | <0.001 |
| Initial T.Bilirubin (mg/dL) |  | 1.098 | 1.054 | 1.143 | <0.001 |
| Pre-OP T. bilirubin (mg/dL) |  | 1.156 | 1.019 | 1.311 | 0.024 |
| Pre-OP PNI |  | 0.961 | 0.921 | 1.003 | 0.066 |
| BMI |  | 0.976 | 0.884 | 1.078 | 0.633 |
| OP time (min) |  | 1.002 | 1.000 | 1.005 | 0.082 |
| Estimated Blood Loss (ml) |  | 1.000 | 0.000 | 1.001 | 0.127 |
| Total LN number |  | 1.009 | 0.990 | 1.028 | 0.362 |
| Positive LN number |  | 1.187 | 1.129 | 1.248 | <0.001 |
| Radiologic tumor size (mm) |  | 1.015 | 0.990 | 1.040 | 0.255 |
| Pathologic tumor size (mm) |  | 1.017 | 0.995 | 1.040 | 0.120 |
| Gender | 1: Male | 1(ref) |  |  |  |
|  | 2: Female | 0.883 | 0.504 | 1.548 | 0.664 |
| Pre-OP Bile drainage | 0: No | 1(ref) |  |  |  |
|  | 1: Yes | 1.453 | 0.812 | 2.598 | 0.208 |
| OP Method | 1: open | 1(ref) |  |  |  |
|  | 2: lapa | 0.183 | 0.025 | 1.323 | 0.092 |
| Transfusion | 0: No | 1(ref) |  |  |  |
|  | 1: Yes | 1.017 | 0.520 | 1.991 | 0.960 |
| Complication | 0: No | 1(ref) |  |  |  |
|  | 1: Yes | 1.128 | 0.626 | 2.032 | 0.688 |
| POPF | 0: No | 1(ref) |  |  |  |
|  | 1: Grade A | 0.817 | 0.398 | 1.677 | 0.582 |
|  | 2: Grade B | 1.379 | 0.652 | 2.916 | 0.400 |
|  | 3: Grade C | 3.635 | 0.487 | 27.127 | 0.208 |
| Residual Cancer Criteria | 0: R0 | 1(ref) |  |  |  |
| (R-status) | 1: R1 | 2.727 | 0.842 | 8.834 | 0.094 |
| Perineural invasion | 0: No | 1(ref) |  |  |  |
|  | 1: Yes | 2.584 | 1.403 | 4.762 | 0.002 |
| Lymphovascular invasion | 0: No | 1(ref) |  |  |  |
|  | 1: Yes | 2.531 | 1.386 | 4.621 | 0.003 |
| Subtype of adenocarcinoma | 1: pancreatobiliary | 1(ref) |  |  |  |
|  | 2: intestinal | 0.226 | 0.115 | 0.444 | <0.001 |
| Tumor gross type | 1: polypoid | 1(ref) |  |  |  |
|  | 2: ulceration | 2.200 | 0.828 | 5.846 | 0.114 $ |
|  | 3: mixed | 2.000 | 0.120 | 33.270 | 0.629 $ |
|  | 4: unknown | 3.667 | 1.220 | 11.021 | 0.021 $ |
| Tumor differentiation grade | 1: well | 1(ref) |  |  |  |
|  | 2: moderate | 3.333 | 1.547 | 7.181 | 0.002 |
|  | 3: poor | 9.910 | 32.05 | 30.639 | <0.001 |
| AJCC8th T stage | 1: IA | 1(ref) |  |  |  |
|  | 2: IB | 15786.3 | 0.000 | 1.246 E+75 | 0.908 |
|  | 3: II | 27052.2 | 0.000 | 2.134 E+75 | 0.902 |
|  | 4: IIIA | 48475.2 | 0.000 | 3.825 E+75 | 0.897 |
|  | 5: IIIB | 89926.6 | 0.000 | 7.092 E+75 | 0.891 |
| AJCC8th N stage | 0: No | 1(ref) |  |  |  |
|  | 1: N1 | 4.281 | 2.141 | 8.563 | <0.001 |
|  | 2: N2 | 9.764 | 4.879 | 19.540 | <0.001 |
| Adjuvant chemotherapy | 0: No | 1(ref) |  |  |  |
|  | 1: Yes | 2.502 | 1.406 | 4.453 | 0.002 |
| PNI_cut off** | 0: PNI<=48.85 | 1(ref) |  |  |  |
|  | 1: PNI>48.85 | 0.544 | 0.283 | 1.045 | 0.068 |
| Initial_CA19_cut off** | 0: initial_CA19-9<53.19 | 1(ref) |  |  |  |
|  | 1: initial_CA19-9≥53.19 | 2.643 | 1.503 | 4.649 | 0.001 |

.

**Table 3. Multivariable Cox regression result**

| **Variables** | **Index level** | **Death (0: survival 1: recur)** | | | |
| --- | --- | --- | --- | --- | --- |
|  |  | **HR** | **Lower** | **Upper** | **p-value** |
| Initial CA19-9 (U/mL) |  | 1.001 | 1.000 | 1.003 | 0.021 |
| Pre-OP T. bilirubin (mg/dL) |  | 1.182 | 1.013 | 1.380 | 0.034 |
| Positive LN number |  | 1.117 | 1.017 | 1.227 | 0.021 |
| Subtype of adenocarcinoma | 1: pancreatobiliary | 1(ref) |  |  |  |
|  | 2: intestinal | 0.292 | 0.136 | 0.625 | 0.002 |

The continuous variables were expressed as the mean ± standard deviation, and the categorical variables were expressed as the frequency (%). Student’s t-test was performed with the continuous variables which were normally distributed, and Mann-Whitney U test used for the continuous variables which were not normally distributed. Chi-square test or Fisher’s extract test was used for the categorical variables.
To evaluate oncologic outcomes and survival analysis, selection of statistically significant variables (p<0.05) was done, following univariate Cox regression test. These variables underwent multivariate Cox regression analysis to evaluate oncologic outcomes. Backward elimination used for final multivariate Cox regression results.
