# Supplementary material for: Preoperative prognostic nutritional index as an independent prognostic factor for resected ampulla of Vater cancer
Source: PLoS One. 2020 Mar 3;15(3):e0229597. doi: 10.1371/journal.pone.0229597 (PMC7053754; doi:10.1371/journal.pone.0229597)
Supplement: S2 File — (DOCX) [file pone.0229597.s002.docx]

**Supplement 2. Statistical analysis of disease free survival (DFS) under PNI Low/High stratification**

**Table 1. Basic characteristics**

- **Cut off value deducted from the Contal and O’quigley’s method

| **Variables** | **Index level** | **0 (Low)**  **(N=80)** | **1 (High)**  **(N=38)** | **p-value** |
| --- | --- | --- | --- | --- |
| Age (Year) |  | 62 (56 – 69) | 59 (51 – 66) | 0.034 |
| Initial CA19-9 (U/mL) |  | 59.6 (36.8 – 82.4) | 100.8 (76.2 – 125.4) | 0.638 |
| Initial T.Bilirubin (mg/dL) |  | 5.52 (1.67 – 9.37) | 2.21 (1.31 – 3.11) | <0.001 |
| Pre-OP T. bilirubin (mg/dL) |  | 2.05 (1.00 - 3.10) | 0.97 (0.67 – 1.27) | 0.001 |
| Pre-OP PNI |  | 42.56±4.53 | 53.66±3.07 | <0.001 |
| BMI |  | 22.86±2.65 | 24.14±2.85 | 0.018 |
| OP time (min) |  | 406 (356 - 457) | 423 (347 - 500) | 0.584 |
| Estimated Blood Loss (ml) |  | 550 (256 - 844) | 538 (263 - 813) | 0.906 |
| Total LN number |  | 23 (15 - 31) | 20 (9 - 31) | 0.235 |
| Positive LN number |  | 2 (1 - 4) | 0 (0 - 0) | 0.132 |
| Radiologic tumor size (mm) |  | 21 (16 - 26) | 20 (15 - 26) | 0.568 |
| Pathologic tumor size (mm) |  | 23 (18 - 28) | 22 (15 - 29) | 0.768 |
| Gender | 1: Male | 47(58.8%) | 17(44.7%) | 0.219 |
|  | 2: Female | 33(41.2%) | 21(55.3%) |  |
| Pre-OP Bile drainage | 0: No | 24(30.0%) | 24(63.2%) | 0.001 |
|  | 1: Yes | 56(70.0%) | 14(36.8%) |  |
| OP Method | 1: open | 77(96.3%) | 32(84.2%) | 0.030 |
|  | 2: lapa | 3(3.7%) | 6(15.8%) |  |
| Transfusion | 0: No | 62(77.5%) | 33(86.8%) | 0.343 |
|  | 1: Yes | 18(22.5%) | 5(13.2%) |  |
| Complication | 0: No | 32(40.0%) | 9(23.7%) | 0.125 |
|  | 1: Yes | 48(60.0%) | 29(76.3%) |  |
| POPF | 0: No | 53(66.3%) | 16(42.1%) | 0.000 |
|  | 1: Grade A | 9(11.2%) | 19(50.0%) |  |
|  | 2: Grade B | 18(22.5%) | 2(5.3%) |  |
|  | 3: Grade C | 0(0%) | 1(2.6%) |  |
| Residual Cancer Criteria | 0: R0 | 76(95.0%) | 38(100.0%) | 0.304 |
| (R-status) | 1: R1 | 4(5.0%) | 0(0%) |  |
| Perineural invasion | 0: No | 64(80.0%) | 34(89.5%) | 0.308 |
|  | 1: Yes | 16(20.0%) | 4(10.5%) |  |
| Lymphovascular invasion | 0: No | 59(73.8%) | 32(84.2%) | 0.303 |
|  | 1: Yes | 21(26.2%) | 6(15.8%) |  |
| Subtype of adenocarcinoma | 1: pancreatobiliary | 49(61.2%) | 14(36.8%) | 0.022 |
|  | 2: intestinal | 31(38.8%) | 24(63.2%) |  |
| Tumor gross type | 1: polypoid | 48(60.0%) | 30(79.0%) | 0.255 |
|  | 2: ulceration | 17(21.3%) | 4(10.5%) |  |
|  | 3: mixed | 2(2.5%) | 0(0%) |  |
|  | 4: unknown | 13(16.2%) | 4(10.5%) |  |
| Tumor differentiation grade | 1: well | 26(32.5%) | 16(42.1%) | 0.535 |
|  | 2: moderate | 50(62.5%) | 20(52.6%) |  |
|  | 3: poor | 4(5.0%) | 2(5.3%) |  |
| AJCC8th T stage | 1: Tis+IA | 4(5.0%) | 5(13.2%) | 0.120 |
|  | 2: IB | 18(22.5%) | 12(31.6%) |  |
|  | 3: II | 20(25.0%) | 12(31.6%) |  |
|  | 4: IIIA | 16(20.0%) | 3(7.8%) |  |
|  | 5: IIIB | 22(27.5%) | 6(15.8%) |  |
| AJCC8th N stage | 0: No | 49(61.2%) | 27(71.1%) | 0.247 |
|  | 1: N1 | 15(18.8%) | 8(21.1%) |  |
|  | 2: N2 | 16(20.0%) | 3(7.8%) |  |
| Adjuvant chemotherapy | 0: No | 42(52.5%) | 27(71.1%) | 0.087 |
|  | 1: Yes | 38(47.5%) | 11(28.9%) |  |
| Recurrence | 0: No | 43(53.8%) | 26(68.4%) | 0.190 |
|  | 1: Yes | 37(46.2%) | 12(31.6%) |  |
| Death | 0: No | 45(56.3%) | 32(84.2%) | 0.006 |
|  | 1: Yes | 35(43.7%) | 6(15.8%) |  |
| Initial_CA19_cut off** | 0: initial_CA19-9<53.19 | 42(52.5%) | 30(78.9%) | 0.011 |
|  | 1: initial_CA19-9≥53.19 | 38(47.5%) | 8(21.1%) |  |

**Table 2. Univariable Cox regression result**

- **Cut off value deducted from the Contal and O’quigley’s method
- ^$^Using firth bias correction for the estimation of 95% CI

| **Variables** | **Index level** | **PNI (0: Low 1: High)** | | | |
| --- | --- | --- | --- | --- | --- |
|  |  | **HR** | **Lower** | **Upper** | **p-value** |
| Age (Year) |  | 1.013 | 0.983 | 1.044 | 0.412 |
| Initial CA19-9 (U/mL) |  | 1.002 | 1.001 | 1.004 | <0.001 |
| Initial T.Bilirubin (mg/dL) |  | 1.090 | 1.045 | 1.138 | <0.001 |
| Pre-OP T. bilirubin (mg/dL) |  | 1.120 | 0.979 | 1.280 | 0.099 |
| Pre-OP PNI |  | 0.983 | 0.915 | 1.055 | 0.631 |
| BMI |  | 0.994 | 0.897 | 1.102 | 0.914 |
| OP time (min) |  | 1.002 | 1.000 | 1.005 | 0.091 |
| Estimated Blood Loss (ml) |  | 1.000 | 1.000 | 1.001 | 0.173 |
| Total LN number |  | 1.009 | 0.990 | 1.028 | 0.340 |
| Positive LN number |  | 1.188 | 1.126 | 1.254 | <0.001 |
| Radiologic tumor size (mm) |  | 1.013 | 0.988 | 1.039 | 0.305 |
| Pathologic tumor size (mm) |  | 1.015 | 0.993 | 1.038 | 0.171 |
| Gender | 1: Male | 1(ref) |  |  |  |
|  | 2: Female | 0.957 | 0.543 | 1.684 | 0.878 |
| Pre-OP Bile drainage | 0: No | 1(ref) |  |  |  |
|  | 1: Yes | 1.272 | 0.698 | 2.315 | 0.431 |
| OP Method | 1: open | 1(ref) |  |  |  |
|  | 2: lapa | 0.216 | 0.029 | 1.590 | 0.132 |
| Transfusion | 0: No | 1(ref) |  |  |  |
|  | 1: Yes | 0.929 | 0.473 | 1.828 | 0.832 |
| Complication | 0: No | 1(ref) |  |  |  |
|  | 1: Yes | 1.287 | 0.706 | 2.342 | 0.410 |
| POPF | 0: No | 1(ref) |  |  |  |
|  | 1: Grade A | 1.084 | 0.501 | 2.347 | 0.837 |
|  | 2: Grade B | 1.300 | 0.614 | 2.754 | 0.493 |
|  | 3: Grade C | 7.115 | 0.800 | 63.288 | 0.078 |
| Residual Cancer Criteria | 0: R0 | 1(ref) |  |  |  |
| (R-status) | 1: R1 | 2.268 | 0.691 | 7.4074 | 0.177 |
| Perineural invasion | 0: No | 1(ref) |  |  |  |
|  | 1: Yes | 2.415 | 1.309 | 4.464 | 0.005 |
| Lymphovascular invasion | 0: No | 1(ref) |  |  |  |
|  | 1: Yes | 2.577 | 1.399 | 4.762 | 0.002 |
| Subtype of adenocarcinoma | 1: pancreatobiliary | 1(ref) |  |  |  |
|  | 2: intestinal | 0.240 | 0.121 | 0.478 | <0.001 |
| Tumor gross type | 1: polypoid | 1(ref) |  |  |  |
|  | 2: ulceration | 1.477 | 0.720 | 3.028 | 0.287 $ |
|  | 3: mixed | 1.551 | 0.208 | 11.581 | 0.669 $ |
|  | 4: unknown | 2.330 | 1.142 | 4.757 | 0.020 $ |
| Tumor differentiation grade | 1: well | 1(ref) |  |  |  |
|  | 2: moderate | 3.007 | 1.386 | 6.527 | 0.005 |
|  | 3: poor | 12.454 | 3.884 | 39.937 | <0.001 |
| AJCC8th T stage | 1: IA | 1(ref) |  |  |  |
|  | 2: IB | 5828.1 | 0.000 | 7.471 E+47 | 0.867 |
|  | 3: II | 9887.6 | 0.000 | 1.266 E+48 | 0.859 |
|  | 4: IIIA | 16729.4 | 0.000 | 2.143 E+48 | 0.851 |
|  | 5: IIIB | 30827.2 | 0.000 | 3.945 E+48 | 0.842 |
| AJCC8th N stage | 0: No | 1(ref) |  |  |  |
|  | 1: N1 | 4.267 | 2.130 | 8.548 | <0.001 |
|  | 2: N2 | 10.557 | 5.202 | 21.423 | <0.001 |
| Adjuvant chemotherapy | 0: No | 1(ref) |  |  |  |
|  | 1: Yes | 2.342 | 1.307 | 4.202 | 0.004 |
| Initial_CA19_cut off** | 0: initial_CA19-9<53.19 | 1(ref) |  |  |  |
|  | 1: initial_CA19-9≥53.19 | 2.415 | 1.339 | 4.367 | 0.003 |

**Table 3. Multivariable Cox regression result**

| **Variables** | **Index level** | **Death (0: survival 1: death)** | | | |
| --- | --- | --- | --- | --- | --- |
|  |  | **HR** | **Lower** | **Upper** | **p-value** |
| Initial CA19-9 (U/mL) |  | 1.002 | 1.000 | 1.003 | 0.021 |
| Subtype of adenocarcinoma | 1: pancreatobiliary | 1(ref) |  |  |  |
|  | 2: intestinal | 0.319 | 0.146 | 0.697 | 0.004 |
| AJCC8th N stage | 0: No | 1(ref) |  |  |  |
|  | 1: N1 | 3.280 | 1.551 | 6.933 | 0.002 |
|  | 2: N2 | 4.744 | 2.178 | 10.333 | <0.001 |

The continuous variables were expressed as the mean ± standard deviation, and the categorical variables were expressed as the frequency (%). Student’s t-test was performed with the continuous variables which were normally distributed, and Mann-Whitney U test used for the continuous variables which were not normally distributed. Chi-square test or Fisher’s extract test was used for the categorical variables.
To evaluate oncologic outcomes and survival analysis, selection of statistically significant variables (p<0.05) was done, following univariate Cox regression test. These variables underwent multivariate Cox regression analysis to evaluate oncologic outcomes. Backward elimination used for final multivariate Cox regression results.
